# Trends in Condom Use and Risk Behaviours after Sexual Exposure to HIV: A Seven-Year Observational Study

**DOI:** 10.1371/journal.pone.0104350

**Published:** 2014-08-26

**Authors:** Enrique Casalino, Christophe Choquet, Agathe Leleu, Romain Hellmann, Mathias Wargon, Gaelle Juillien, Yazdan Yazdanpanah, Elisabeth Bouvet

**Affiliations:** 1 Assistance Publique-Hôpitaux de Paris (AP-HP), University Hospital Bichat-Claude Bernard, Emergency Department, Paris, France; 2 Université Paris Diderot, Sorbonne Paris Cité, EA 7334 «Recherche clinique coordonnée ville-hôpital, Méthodologies et Société (REMES)», Paris, France; 3 Study Group for Efficiency and Quality of Emergency Departments and Non-Scheduled Activities Departments, Paris, France; 4 Hôpital Saint Camille, Bry-sur-Marne, France; 5 Assistance Publique-Hôpitaux de Paris (AP-HP), University Hospital Bichat-Claude Bernard, Infectious and Tropical Diseases Department-AIDS Clinic, Paris, France; Alberta Provincial Laboratory for Public Health/University of Alberta, Canada

## Abstract

**Objective:**

We aimed to determine the trends in numbers and percentages of sexually exposed persons to HIV (SE) consulting an ED for post-exposure prophylaxis (PEP), as well as predictors of condom use.

**Study Design:**

We conducted a prospective-observational study.

**Methods:**

We included all SE attendances in our Emergency Department (ED) during a seven-year study-period (2006–2012). Trends were analyzed using time-series analysis. Logistic Regression was used to define indicators of condom use.

**Results:**

We enrolled 1851 SE: 45.7% reported intercourse without condom-use and 12.2% with an HIV-infected partner. Significant (p<0.01) rising trends were observed in the overall number of SE visits (+75%), notably among men having sex with men (MSM) (+126%). There were rising trends in the number and percentage of those reporting intercourse without condom-use in the entire population +91% (p<0.001) and +1% (p>0.05), in MSM +228% (p<0.001) and +49% (p<0.001), in Heterosexuals +68% (p<0.001) and +10% (p = 0.08). Among MSM, significant rising trends were found in those reporting high-risk behaviours: anal receptive (+450% and +76%) and anal insertive (+l33% and +70%) intercourses. In a multivariate logistic regression analysis, heterosexuals, vaginal intercourse, visit during the night-shift and short time delay between SE and ED visit, were significantly associated with condom-use.

**Conclusion:**

We report an increasing trend in the number of SE, mainly among MSM, and rising trends in high-risk behaviours and unprotected sexual intercourses among MSM. Our results indicate that SE should be considered as a high-risk population for HIV and sexually transmitted diseases.

## Introduction

Post-exposure prophylaxis (PEP) has been recommended around the world to prevent HIV infection following a high risk sexual encounter [1], [2]. Even if PEP effectiveness is not proven and its use remains controversial [3], [4], it is currently accepted that PEP represents a medical and therapeutic emergency condition, and that PEP should be proposed as soon as possible [5].

Surveys on sexual behaviour and condom use have focused on sexual and preventive practices among the general population [6] and men having sex with men (MSM) [7], some of them reporting a substantial increase in condom use before 2000 [8]. In France, a repeated survey across the general population indicates that the proportion of individuals reporting condom use at their most recent intercourse in 2010 is at its lowest reported level during an 18 year follow-up period [6]. Furthermore, the high incidence and the rising trend in sexually transmitted diseases (STD), notably among MSM [9], indicate that unprotected sexual activity remains a health concern.

In France, PEP is available free of charge in all Emergency Departments (ED) for occupational and non-occupational sexual exposures. In some other countries, ED initiates PEP after sexual exposure [10]. It has been suggested that patients that are sexually exposed to HIV patients are risk takers and have an increased risk of HIV-infection [11]. Nevertheless, no study has described, among sexually exposed patients to HIV, their behaviours and condom use.

The primary objective of the present study was to determine the trends in the number and the percentage of those reporting sexual intercourse without condom use among patients consulting an ED after a sexual exposure to HIV. The second objective was to determine the characteristics associated with condom use.

## Methods

### Study design

This was an observational prospective study.

### Setting and study period

The study was conducted in a university hospital located in Paris metropolitan area. Our ED treats more than 70 000 patients each year. The hospital includes an Infectious and tropical diseases-AIDS Clinic unit that cares for more than 4000 HIV-infected patients.

The study period was from January 1, 2006 to December 31, 2012 (seven-year study period).

### Selection of participants

All patients consulting the ED after sexual exposure to HIV were included.

### Ethics Statement

This dataset was completely anonymous and did not contain any identifiable personal health information. The dataset is currently used as an ED quality measure of PEP prescription as part of an ongoing emergency activity and performance evaluation.

This study has been approved by the Emergency Committees on Ethics, Research and Informatics (Assistance Publique-Hôpitaux de Paris).

### Methods and measurements

Sexual exposure characteristics were systematically reported in the ED. They were mandatory to prescribe and obtain PEP. Studied variables: age; sex; condom use (no, yes); HIV status of sexual partner; type (MSM, heterosexual); sexual intercourse (anal, vaginal, oral; insertive, receptive) forming six Groups: i) Anal Receptive (AR); ii) Anal Insertive (AI); iii) Vaginal Receptive (VR); iv) Vaginal Insertive (VI); v) Oral Receptive (OR); vi) Oral Insertive (OI). According to our triage scale that indicates the waiting time before seeing an ED physician (WT-to-P), triage for potential HIV-exposures (occupational or sexual) is based on the time delay between exposure to HIV and the arrival in ED: level 2 <12 hours (WT-to-P <20 minutes); level 3 <48 hours (WT-to-P <60 minutes); level 4 ≥48 hours (WT-to-P <120 minutes).

The number of sexual exposures, and number and percentage of those reporting sexual intercourse without condom use were calculated by trimester.

### Statistical Analysis

A chi-squared test for trend was conducted to identify any significant change over study period for data collected on annual basis. Trends in the numbers and percentages of sexual exposures to HIV and among those reporting sexual intercourse without condom use, obtained as trimester values, were first evaluated by logistic or linear regressions, with trimesters (28 trimesters) as the sole explanatory variable. Then we used time series-analysis (autoregressive integrated moving average (ARIMA) models) using Box–Jenkins methodology [12]. Time series methods can diagnose the precise nature of the correlation and adjust it. Moving averages provide a useful way of presenting time series data, highlighting any long-term trends whilst smoothing out any short-term fluctuations. The efficiency of time series methods may give in a steeper slope than that generated by Poisson regression [13]. In brief, data was transformed if necessary to eliminate trend. The model was identified by determining the ARIMA model orders (p, d, q) using autocorrelation and partial autocorrelation, then the adequacy of the model was checked and statistical significance of the parameters was determined. These models refer to a complex process that incorporates information from past observations and past errors with the observations into the estimation of predicted values.

To assess the association between condom use and studied variables we used logistic regression. Variables that showed near statistical significance (p<0.1) were included in the multivariate stepwise logistic regression model to determine those independently related to end-points.

Statistica 10® (StatSoft) software was used for data collection and analysis.

## Results

### Characteristics of the study population

We enrolled 1851 sexual exposures to HIV. Main characteristics of the entire population were: age (years) 32.2±9 (average 32); males 1408 (76%), females 443 (23.9%); MSM 788 (42.6%), Heterosexuals 1063 (57.4%); HIV positive status of sexual partner 232 (12.5%), negative or unknown 1619 (87.5%); Condom use: no 845 (45.7%), yes 1006 (54.3%); Arrival time to ED: 8am to 6pm: 768 (41.8%), 6pm to noon: 680 (37%), and noon to 8am: 391 (21.3%); Delay between SE and arrival in ED: <12 h: 1022 (56.1%), 12 h to 48 h: 497 (27.3%), >48 h: 303 (16.6%); Type of intercourse AR 442 (23.9%), AI 314 (17%), VR 393 (21.2%), VI 537(29%), OR 105 (5.7%), OI 51 (2.8%), Other/Unknown 9 (0.5%).

PEP was prescribed for 1349 (72.9%).

As shown in Table 1, we find significant increasing trend over the study period in terms of MSM visits and sexual partners known to be HIV-positive.

**Table 1:** Main characteristics of the study population as a function of year.

	2006 n (%)	2007 n (%)	2008 n (%)	2009 n (%)	2010 n (%)	2011 n (%)	2012 n (%)	P*
Sex								0.1
Female	46 (24.5)	69 (28)	64 (25.3)	42 (21)	74 (25.5)	76 (22.4)	72 (21.6)	
Male	142 (75.5)	177 (72)	189 (74.7)	158 (79)	216 (74.5)	264 (77.6)	262 (78.4)	
Sexual type								0.01
MSM	71 (37.8)	101 (41.1)	95 (37.6)	78 (39)	142 (49)	147 (43.2)	154 (46.1)	
Heterosexual	117 (62.2)	145 (58.9)	158 (62.4)	122 (61)	148 (51)	193 (56.8)	180 (53.9)	
Sexual partner known to be HIV positive (*yes*)	18 (9.6)	26 (10.6)	25 (9.9)	24 (12)	39 (13.4)	48 (14.2)	52 (15.6)	<0.001
Details of sexual intercourse								
All patients (n = 1851)								
AR	31 (16.5)	74 (30.1)	65 (25.7)	33 (16.5)	82 (28.3)	77 (22.7)	80 (24)	0.7
AI	36 (19.2)	28 (11.4)	29 (11.5)	39 (19.5)	57 (19.7)	65 (19.1)	60 (18)	<0.0001
VR	42 (22.3)	55 (22.4)	61 (24.1)	38 (19)	70 (24.1)	68 (20)	59 (17.7)	0.1
VI	62 (33)	69 (28.1)	87 (34.4)	70 (35)	57 (19.7)	96 (28.2)	96 (28.7)	0.09
OR	7 (3.7)	13 (5.3)	6 (2.4)	14 (7)	13 (4.5)	20 (5.9)	32 (9.6)	0.004
OI	5 (2.7)	7 (2.9)	5 (2)	3 (1.5)	11 (3.8)	13 (3.8)	7 (2.1)	0.9
MSM (n = 788)								
AR	30 (42.3)	63 (62.4)	63 (66.3)	29 (37.2)	78 (54.9)	73 (49.7)	75 (48.7)	0.2
AI	31 (43.7)	25 (24.8)	24 (25.3)	32 (41)	48 (33.8)	54 (36.7)	53 (34.4)	0.5
OR	6 (8.5)	10 (9.9)	5 (5.3)	14 (18)	13 (9.2)	16 (10.9)	24 (15.6)	0.1
OI	3 (4.2)	3 (3)	2 (2.6)	3 (2.1)	3 (2)	3 (2)	2 (1.3)	0.2
Heterosexuals (n = 1063)								
AR	1 (0.9)	11 (7.6)	2 (1.3)	4 (3.3)	4 (2.7)	4 (2.1)	5 (2.8)	0.4
AI	5 (4.3)	3 (2.1)	5 (3.2)	7 (5.7)	9 (6.1)	11 (5.7)	7 (3.9)	0.3
VR	42 (35.9)	55 (37.9)	61 (38.6)	38 (31.2)	70 (47.3)	68 (35.2)	59 (32.8)	0.6
VI	62 (53)	69 (47.6)	87 (55.1)	70 (57.4)	57 (38.5)	96 (49.7)	96 (53.3)	0.7
OR	1 (0.9)	3 (2.1)	1 (0.6)	0 (0)	0 (0)	4 (2.1)	8 (4.4)	0.03
OI	2 (1.7)	4 (2.8)	2 (1.3)	1 (0.8)	8 (5.4)	10 (5.2)	5 (2.8)	0.09

* Chi-squared for trend. For sexual intercourse details, each group was compared to all the other sexual intercourses.

By using time-series analysis, significant rising trends were observed in the overall number of attendances (+75%; p<0.0001), MSM (+126%; p<0.0001) and Heterosexuals (+46%; p<0.001).

### Trends in the number and percentage of patients reporting sexual intercourse without condom use

**Figure 1:**
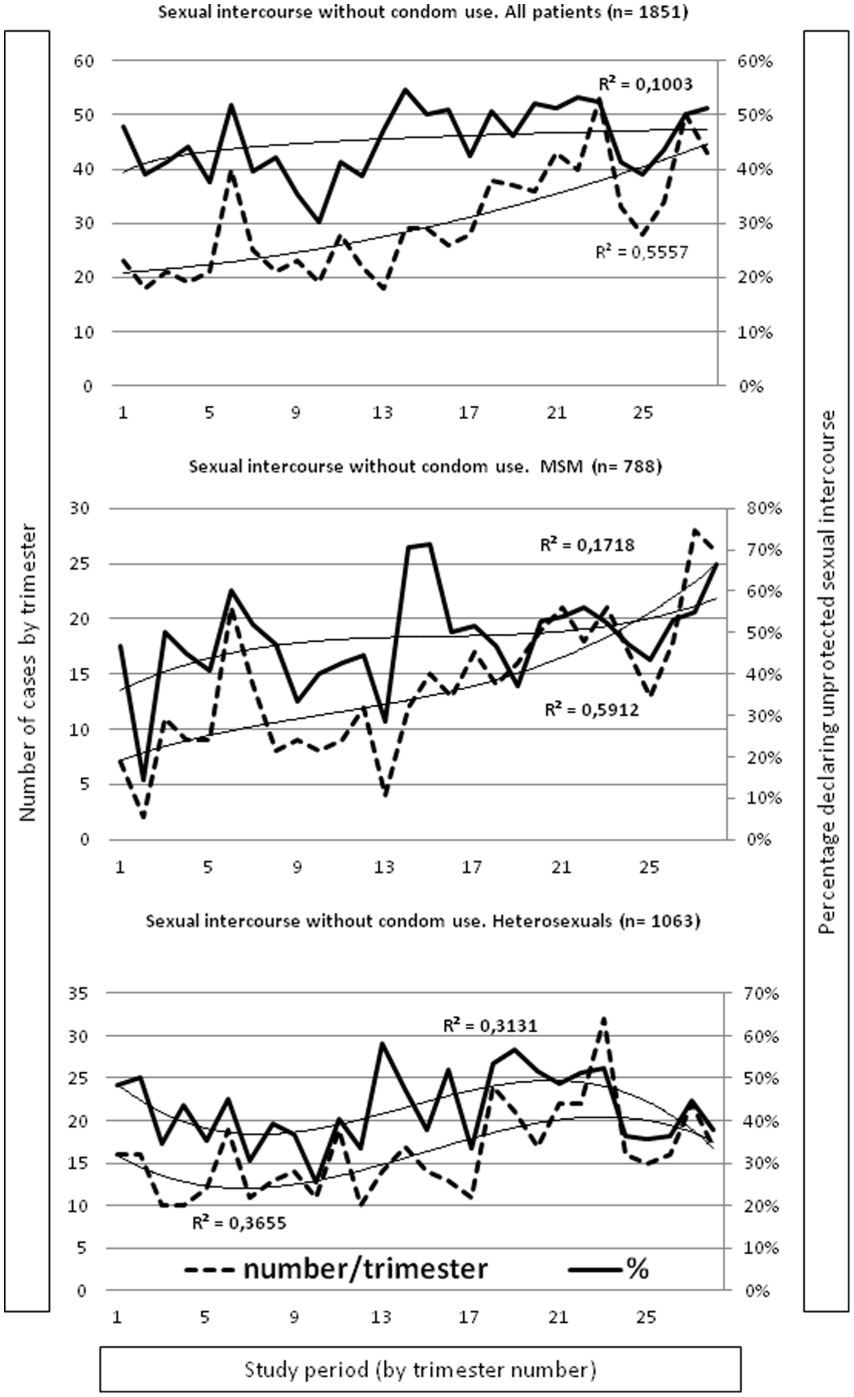
Trends in numbers and percentages of sexual intercourse without condom use.

As presented in Fig. 1, a significant increase in the number of attendances reporting sexual intercourse without condom use (+91%; p<0.001) was found for every kind of population but not in the percentage of those reporting sexual intercourse without condom use (+1%; p>0.05). Among MSM, we observed significant increasing trends in both number (+228%; p<0.001) and percentage (+49%; p<0.001). Among heterosexuals, we found a significant increasing trend in the number (+68%; p<0.001) but not in the percentage (+10%; p = 0.08).

**Figure 2:**
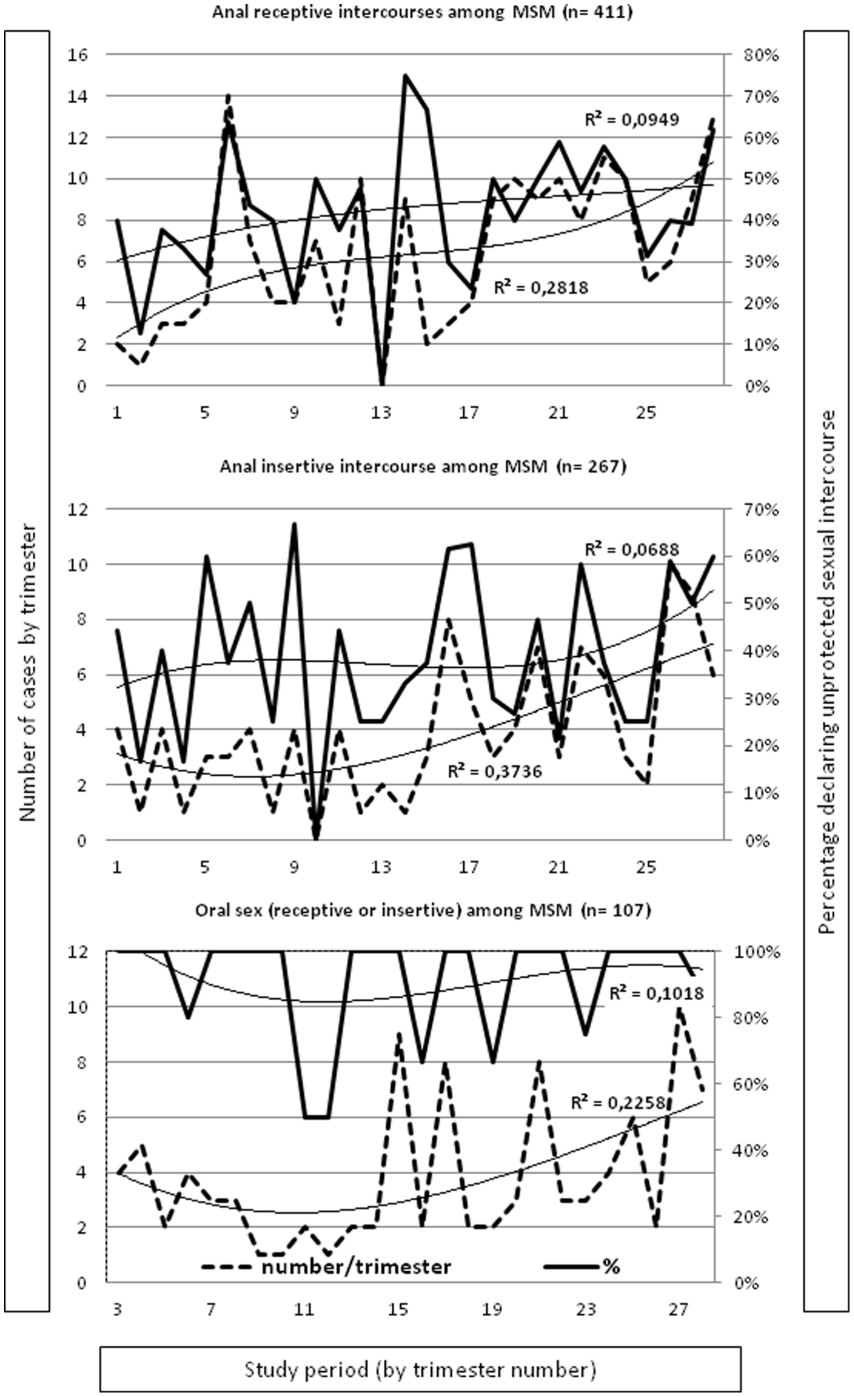
Trends in numbers and percentages of sexual intercourse without condom use among MSM.

Among MSM (Fig. 2), there were significant increasing trend in the number (+450, p<0.0001) and percentage (+76, p<0.01) of AR intercourses without condom use. Similar features were observed for AI intercourses (+133% (p<0.0001) and +70 (p<0.001)). The number of oral intercourses (receptive and insertive) without condom use significantly increased over the study period (+71%; p<0.01) although the percentage of attendances reporting oral sex without condom use remained stable, with values above 95%.

**Figure 3:**
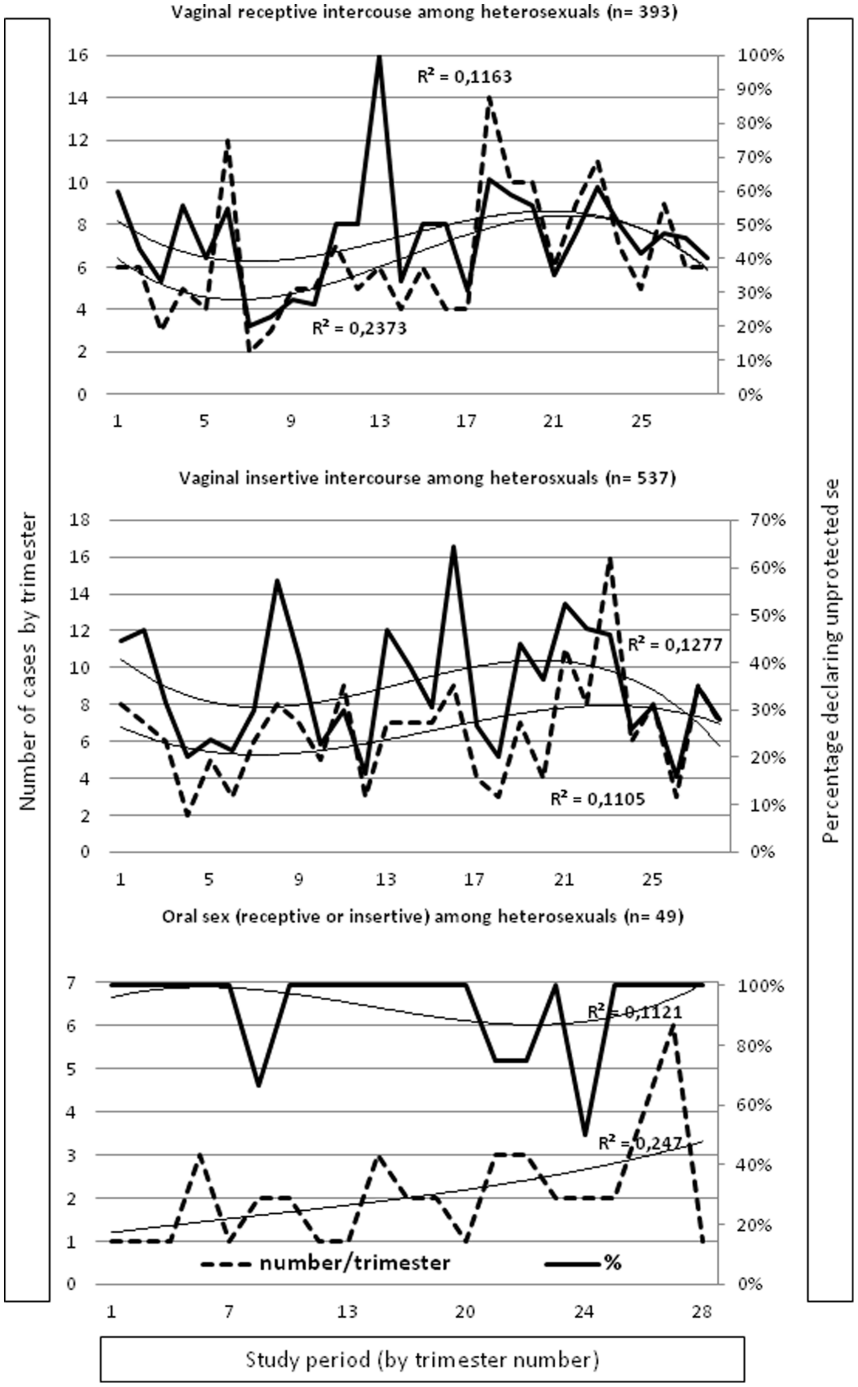
Trends in numbers and percentages of sexual intercourse without condom use among Heterosexuals.

Among heterosexuals (Fig. 3), there were no significant trend in the numbers or percentages of VR (+2% (p = 0.1) and −8% (p = 0.09)) and VI intercourses without condom use (−1% (p = 0.9) and −48% (p = 0.05)), respectively. For Oral intercourses without condom use (OR plus OI) we found a significant increasing trend in the number (+166; p<0.001) but not in the percentage of those reporting intercourse without condom use (−1%, p = 1).

### Predictors for condom use

Overall, 1006/1851 (54.3%) reported condom use. Table 2 presents the results of multivariate logistic regression analysis for protected sexual intercourse.

**Table 2:** Predictors of protected sexual intercourse (n = 1851).

	Unadjusted Logistic Regression			Multivariate Logistic Regression	
	Condom use		P	OR (95% CI)	P
	No (n (%))	Yes (n (%))			
Age groups			0.9		
<20	35 (46.7)	40 (53.3)			
≥20-<30	387 (46.6)	444 (53.4)			
≥30-<40	272 (44.2)	343 (55.8)			
≥40	151 (45.8)	179 (54.2)			
Sex			0.2		
Female	214 (48.3)	229 (51.7)			
Male	631 (44.8)	777 (55.2)			
Sexual type			0.003		<0.0000001
MSM	391 (49.6)	397 (50.4)		1	
Heterosexual	454 (42.7)	609 (57.3)		1.32 (1.07–1.63)	
Year			0.01		
2006	81 (43.1)	107 (56.9)			
2007	107 (43.5)	139 (56.5)			
2008	92 (36.4)	161 (63.6)			
2009	102 (51)	98 (49)			
2010	139 (47.9)	151 (52.1)			
2011	169 (49.7)	171 (50.1)			
2012	155 (46.4)	179 (53.6)			
Arrival time to ED			0.005		0.006
8 am to 6 pm	372 (48.4)	396 (51.6)		1	
6 pm to noon	316 (46.5)	364 (53.5)		1.2 (1.05–1.36)	
Noon to 8 am	151 (38.6)	240 (61.4)		1.43 (1.36–1.63)	
Time delay nO-SE to ED			0.00006		0.000002
<12 h	424 (41.5)	598 (60.5)		1	
12 to 48 h	243 (48.9)	254 (51.1)		0.76 (0.67–0.86)	
>48 h	166 (54.8)	137 (45.2)		0.57 (0.51–0.65)	
Sexual intercourse details					
Oral	143 (91.7)	13 (8.3)	<0.00001	1	<0.000001
Anal	330 (43.7)	426 (53.3)		4.36 (3.24–5.86)	
Vaginal	365 (39.3)	565 (60.7)		19 (14.2–25.6)	
Receptive	376 (45)	459 (55)	<0.000001		
Insertive	319 (37.5)	532 (62.5)			
AR	196 (44.3)	246 (55.7)	<0.000001		
AI	134 (42.7)	180 (57.3)			
VR	180 (45.8)	213 (54.2)			
VI	185 (34.5)	352 (65.6)			
OR	97 (92.4)	8 (7.6)			
OI	46 (90.2)	5 (9.8)			
HIV status of sexual partner			0.5		
Positive	43 (49.4)	44 (50.6)			
Unknown or negative	438 (45.6)	523 (54.4)			
PEP prescribed in the ED			0.4		
no	216 (48.1)	233 (51.9)			
yes	604 (44.8)	745 (55.2)			
no data available	25 (47.2)	28 (52.8)			

## Discussion

In our study, potentially sexually exposed patients to HIV were young, mainly men, and some of them were heterosexuals. Out of interest, 17% of them arrived in the ED beyond 48 hours, thus limiting PEP prescription. Unprotected sexual intercourses achieved 45.7% and high-risk behaviours 62.1% (AR, AI and VR), and 12.5% reported knowing their sexual partner being HIV-infected. Moreover, our study highlights significant increases in the overall number of attendances both for MSM and heterosexuals, but we can observe significant increasing trends in the number and percentage of high-risk behaviours and unprotected intercourses only among MSM. All these features may explain the 73% PEP-prescription rate. This indicates that sexually exposed patients, notably MSM, are at high-risk for HIV and other sexually transmitted diseases.

It has been reported that the numbers go up to 32% among sexually exposed patients having intercourse with positive HIV-partners [4]. In the present study, we show a 12% mean rate with a significant increasing trend over the study period. HIV-prevalence in the Paris metropolitan area is estimated to be 0.5%, but among MSM it may be up to 18% [14].

Similarly, our study shows rising trends in the number of sexual exposures to HIV attendances (+75%), higher among MSM (+126%) than among heterosexuals (+46%). Only two studies have previously focused on sexual exposures to HIV frequency, one reporting a slight increase in the numbers in Spain [15] and the second reporting a +850% over a ten-year period in Switzerland [16].

In our study, the percentage of those reporting sexual intercourse without condom use is higher than previously reported in the general population, MSM and heterosexuals [6], [17]. Nevertheless, the studies evaluating the use of a condom during the last intercourse, a currently accepted indicator [18], show the same kind of results as ours [19]. We can observe a significant rise in the numbers of MSM and heterosexuals reporting sexual intercourse without condom use. However, we can only notice among MSM a significant increase in the percentage of those reporting sexual intercourse without condom use (+46%). A recently published survey indicated a significant reduction in condom use among the general French population [6].

Nevertheless we can't notice a significant decrease in the percentage of heterosexuals reporting VR and VI unprotected intercourses, we found significant increases in the number but also in the percentage of MSM reporting AR and AI unprotected intercourses. Anal intercourse is recognized as a high-risk behaviour for HIV transmission [20]. The increase in rates for sexually transmitted diseases in the general population and mainly among MSM [21], and the 22% increase in the number of HIV reported cases among MSM in Europe [22], indicate a relapse in sexually transmitted diseases and HIV prevention strategies.

When we established the factors associated with condom use, we found on a multivariate analysis that MSM use condoms less frequently than heterosexuals and that anal intercourse is less frequently associated with condom use compared to vaginal intercourse. Interestingly, sexual intercourse with a partner known to be HIV infected is not associated with condom use, even in an unadjusted analysis. Our results also indicate that there is a less frequent use of condoms in well recognized [23] high-risk sexual practices. Similarly, we observe that condom use is significantly associated with a rapid ED visit or a visit during the night shift in the ED. We hypothesized that these two features indicate PEP knowledge. Our results indicate that links may exist between information about PEP availability and the use of the condom. It has been reported that increased knowledge in the population and an easy access to health care are factors that increase the uptake of PEP [24]–[26]. In previous studies in the US and in the UK, awareness ranged from 36% to 56% among MSM [24], [27], [28]. In France, 70% of individuals reported the knowledge of PEP, mainly MSM [29]. It has been recently reported that awareness of PEP increases with the number of' previous HIV tests [30]. Between 2008 and 201l, HIV prevention campaigns were conducted in France, including HIV-testing for the general population as well as for those with high risk behaviours.

Several limitations must be considered in interpreting our results. Firstly, this is a single ED study including patients demanding PEP after a potential sexual exposure to HIV. Secondly, we conducted an observational study questioning patients about their last sexual intercourse to define the need for PEP. Patients informed about PEP indications can modify their answers in order to obtain PEP. Thirdly, this study is not a survey on sexual behaviours. To our knowledge, this is the largest and longest over a period of time observational study on patients consulting after potential sexual exposure to HIV, and the first one to describe the use of condoms and the behavioural risk and their trends over a long study period.

### Conclusions

Our results indicate an increase in the number of attendances after sexual exposure to HIV particularly among MSM, but above all an increase of the number and the percentage of HIV high-risk transmission intercourses and unprotected intercourses among MSM. We found that more than half of our study population reported high-risk behaviours and unprotected intercourses, that some of them arrived too late to begin PEP according to French recommendations (48 hours after SE) [6], and that there is an association between PEP awareness and condom use. Thus, we think that potentially sexually exposed persons to HIV should be considered as a high-risk population that may need to be prioritized to receive prevention counselling as a part of PEP comprehensive care. Even if pre-exposure prophylaxis appears as a promising new HIV-prevention strategy [31], [32], our study indicates that awareness and accessibility to PEP must be considered again as one of the HIV-prevention tools.
